# Minimal Change Disease as a Secondary and Reversible Event of a Renal Transplant Case with Systemic Lupus Erythematosus

**DOI:** 10.1155/2015/987212

**Published:** 2015-08-13

**Authors:** Elena Gkrouzman, Kyriakos A. Kirou, Surya V. Seshan, James M. Chevalier

**Affiliations:** ^1^University of Connecticut Health Center, 263 Farmington Avenue, Farmington, CT 06030-1235, USA; ^2^Division of Rheumatology, Hospital for Special Surgery, 535 East 70th Street, New York, NY 10021, USA; ^3^New York-Presbyterian Hospital, 525 East 68th Street, Starr Pavilion 1009, New York, NY 10021, USA; ^4^Rogosin Kidney Center, 505 East 70th Street, New York, NY 10021, USA

## Abstract

Secondary causes of minimal change disease (MCD) account for a minority of cases compared to its primary or idiopathic form and provide ground for consideration of common mechanisms of pathogenesis. In this paper we report a case of a 27-year-old Latina woman, a renal transplant recipient with systemic lupus erythematosus (SLE), who developed nephrotic range proteinuria 6 months after transplantation. The patient had recurrent acute renal failure and multiple biopsies were consistent with MCD. However, she lacked any other features of the typical nephrotic syndrome. An angiogram revealed a right external iliac vein stenosis in the region of renal vein anastomosis, which when restored resulted in normalization of creatinine and relief from proteinuria. We report a rare case of MCD developing secondary to iliac vein stenosis in a renal transplant recipient with SLE. Additionally we suggest that, in the event of biopsy-proven MCD presenting as an atypical nephrotic syndrome, alternative or secondary, potentially reversible, causes should be considered and explored.

## 1. Introduction

Minimal change disease (MCD) is a disease of the podocyte that manifests with sudden onset nephrotic syndrome. Isolated diffuse effacement of the epithelial foot processes on electron microscopy is the defining feature of MCD. Clinically, it is characterized by the development of massive proteinuria, hypoalbuminemia, edema, and hyperlipidemia.

Although the majority of patients have idiopathic or primary MCD, some may exhibit MCD secondary to another disease process or exposure to drugs. Examining patients with secondary MCD allows us to investigate shared mechanisms of pathogenesis [1]. Herein, we report a lupus patient with a renal transplant who developed* de novo* MCD associated with right external iliac vein stenosis.

## 2. Case Presentation

A 27-year-old Latina woman received a living related transplant from her mother for end stage renal disease (ESRD) secondary to advanced lupus nephritis and presented with nephrotic range proteinuria 6 months after transplantation.

The patient was diagnosed with systemic lupus erythematosus (SLE) at age of 17. During the course of the disease she fulfilled the American College of Rheumatology (ACR) criteria including malar rash, arthritis, pericarditis, class IV/V lupus nephritis, leucopenia, lymphopenia, and positive antinuclear (ANA) and anti-double-stranded DNA (anti-dsDNA) antibodies. The patient's lupus nephritis was treated with glucocorticoids, multiple doses of cyclophosphamide, mycophenolate mofetil, and rituximab. Despite aggressive treatment, she progressed to ESRD and required renal transplantation. She received a kidney from her mother, which was donor/recipient CMV +/−. The patient has been negative for anti-Ro, anti-La, anti-Sm, anti-RNP, *β*2 glycoprotein I, and lupus anticoagulant. The anticardiolipin antibody (ACLA), although reported as positive at one occasion which prompted the use of aspirin for prophylaxis, has been consistently negative ever since. She also has a history of hypertension and hypothyroidism due to Hashimoto's thyroiditis.

**Figure 1:**
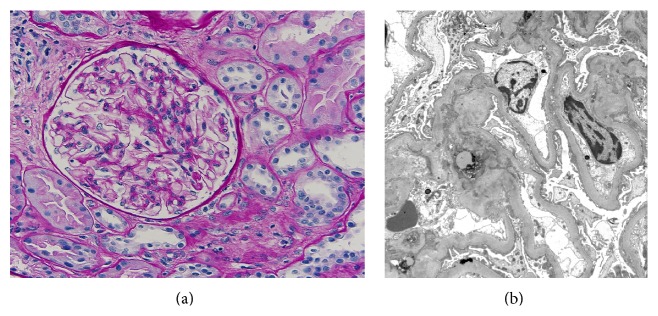
(a) Kidney biopsy with renal cortex showing a normal glomerulus with preserved architecture and mostly preserved tubules (×400, PAS stain). (b) Electron micrographs of glomerulus showing greater than 50% foot process effacement and normal thickness of capillary basement membranes without immune deposits (×6740).

**Figure 2:**
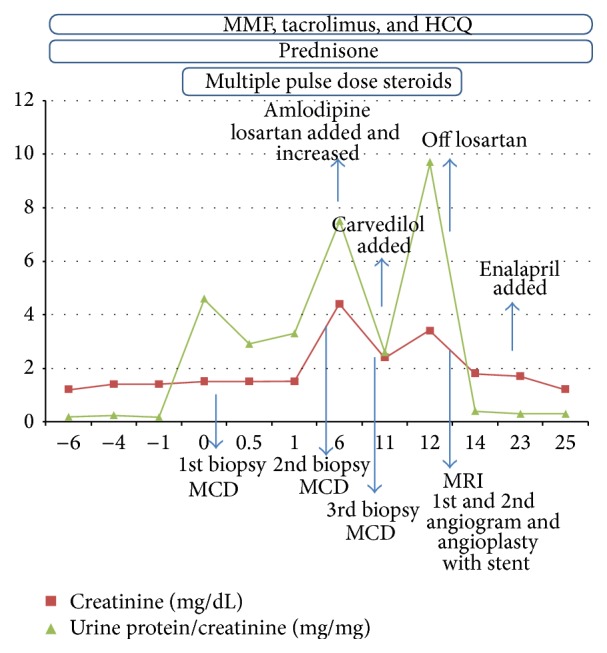
Serum creatinine and urine protein/creatinine ratio over time along with interventions and medical treatments. Time is noted in months where “0” indicates the onset of proteinuria and renal failure, which occurred approximately 6 months after renal transplantation. Patient's proteinuria improved significantly after the 2nd angioplasty (month 12) along with her serum creatinine. MMF: mycophenolate mofetil, HCQ: hydroxychloroquine, MCD: minimal change disease, and MRI: magnetic resonance imaging.

The surgery and hospital course after transplantation were uneventful and the patient was discharged with excellent urine output and creatinine level of 1.0 mg/dL. Six months later she developed nephrotic range proteinuria at 4.6 mg/mg (Table 1) and her creatinine levels progressively rose to 2.5 mg/dL. However, she lacked edema, hypoalbuminemia, and hypercholesterolemia. Her medications at the time included tacrolimus, 4 mg in the morning and 3 mg in the evening, mycophenolate mofetil 500 mg bid, prednisone 5 mg/d, hydroxychloroquine 200 mg bid, enalapril 10 mg/d, aspirin 81 mg/d, trimethoprim-sulfamethoxazole 400–80 mg/d, valganciclovir HCl 450 mg/d, levothyroxine 0.15 mg/d, and omeprazole 20 mg/d. A kidney biopsy was performed that showed minimal glomerular change by light microscopy and diffuse podocyte foot process effacement by electron microscopy, consistent with MCD (Figures 1(a) and 1(b)). There was no evidence of acute cellular, antibody-mediated, or vascular rejection or immune-complex-mediated glomerulonephritis. In particular, immunofluorescence exam did not reveal significant glomerular staining for IgG, IgA, and C3. There was granular glomerular staining in mesangial regions and capillary loops for 1+ IgM while the tubulointerstitial compartment was negative for immunoglobulins or complement components. Arteries and arterioles stained 1+ for C3 but peritubular capillaries were negative for C3d and C4d. The serum C3 and C4 levels were within the normal range and anti-dsDNA antibody was 1+. Consequently, she received pulse methylprednisolone followed by oral prednisone to treat presumed MCD. She did not respond to treatment, while two additional subsequent renal biopsies were also consistent with MCD. In the following 12 months the patient developed several episodes of acute renal failure characterized by increasing proteinuria and creatinine levels that were attributed to dehydration. Her serum albumin levels decreased down to 2.7 g/dL secondary to ongoing protein loss in urine. However, this effect was transient and coincided with one of the peaks of her proteinuria (7.5 g). Otherwise, her albumin during this course usually ranged between 3 and 4 g/dL. Multiple ultrasounds of the kidney were performed. Initially they were normal showing patent renal artery and vein with good perfusion of the transplant on color and power Doppler. However, subsequently the studies exhibited mild hydronephrosis of the transplant kidney and turbulence in the right external iliac vein proximal to the attachment of the transplanted renal vein that was suggestive of narrowing. Eventually, because of worsening proteinuria and creatinine (9.7 mg/mg and 3.4 mg/dL, resp.), magnetic resonance imaging (MRI) of the pelvis without contrast was done for further investigation and revealed diminished signal intensity in the right iliac artery. A subsequent angiogram revealed a normal right iliac artery and renal transplant arterial anastomosis but outlined stenosis of the right external iliac vein adjacent to the transplant anastomosis with a pressure gradient of 7 mmHg between the common iliac vein and the external iliac vein. Balloon angioplasty of the right external iliac vein partially corrected the stenosis with a postangioplasty gradient of 2-3 mmHg. A few weeks later recurrent renal dysfunction necessitated a second venogram that showed restenosis and culminated in angioplasty and stent placement with pressure gradient improving from 10 mmHg to 0. After the stent deployment and dilation to 12–14 mm in diameter, a thrombus was identified within the stent which was aspirated. There was no thrombus seen prior to stenting which leads to the assumption that this was a procedure-related complication. Subsequently, the patient received one month of renally dosed enoxaparin as prophylaxis to decrease the possibility of in-stent thrombosis. Following the procedure, the proteinuria and creatinine levels steadily declined and reached her baseline of 300 mg/day and 1.5 mg/dL, respectively, at one-year follow-up (Figure 2).

## 3. Discussion

Our patient is a unique case of iliac vein stenosis mimicking a podocytopathy similar to* de novo* MCD. Her disease occurred 6 months after transplantation and was characterized by unresponsiveness to high-dose glucocorticoids but immediate and sustained remission of proteinuria and renal failure with restoration of normal venous blood flow at the anastomosis. We hypothesize that the mechanical forces generated by the increase in venous blood pressure at the level of the glomerular tuft due to the iliac vein stenosis may have inflicted or predisposed the foot process effacement of the podocytes covering the glomerular basement membrane (GBM) and resulted in the appearance of MCD on renal biopsy and proteinuria. Our hypothesis may be supported by the fact that two doses of pulse methylprednisolone achieved only partial remission and a subsequent course of oral prednisone did not prevent the development of acute renal failure with persistently increased proteinuria and serum creatinine levels. Nevertheless, once the iliac vein stenosis was visualized and repaired, normal renal function was recovered.

Podocytes are highly specialized cells of epithelial origin that attach to the GBM through their foot processes. These foot processes interdigitate with one another and the filtration slits that are created between them are covered with an extracellular structure, the slit diaphragm [2]. The latter serves as a size- and charge-selective barrier of macromolecule filtration establishing selective permeability [3], while the foot processes with their contractile system stabilize the GBM and counteract local elastic distension caused by high capillary pressures [4]. Our hypothesis is based on observations suggesting that podocytes may respond to stress caused by increases in intracapillary pressures or exposure to toxins with foot process effacement and rearrangement of their actin cytoskeleton [5–7]. This process has been associated temporally with the emergence of proteinuria [8].

In order to explore the consequences of renal vein stenosis on the kidney and whether it has been previously associated with MCD in other instances, we examined cases of the “nutcracker syndrome.” This syndrome is characterized by anatomical stenosis of the left renal vein inflicted by its compression between the aorta and proximal superior mesenteric artery. The left renal vein stenosis, which sometimes can be intermittent, causes congestion of the left kidney and leads to the formation of collateral veins. The clinical characteristics of this syndrome include flank pain, hematuria [9–11], and proteinuria [12–15], particularly of orthostatic type, on urine analysis. The onset of proteinuria in this syndrome may demonstrate a similar mechanism of mechanical forces developing at the glomerulus due to renal or iliac vein stenosis in the posttransplant period that can act as a stress signal to the podocytes and cause foot process retraction in order to avoid further damage [6, 7]. Renal biopsy findings by electron microscopy in nutcracker syndrome complicated with proteinuria did not exhibit podocyte foot process effacement, although the small number of case reports with available electron microscopy reports and the intermittent nature of renal vein compression do not allow us to draw any definite conclusions [16, 17].

The exact pathogenesis of idiopathic MCD has not been fully elucidated. It has been suggested that a dysfunction of cell-mediated immunity, namely, an abnormal clone of T-cells, may result in the production of a cytokine or a circulating factor that alters the glomerular filtration barrier permeability and culminates in foot process effacement and proteinuria [18]. While the molecular structure of this factor remains yet to be discovered, additional data derived from case reports on the effectiveness of rituximab (a chimeric monoclonal antibody against CD20 on the surface of B cells) in steroid-dependent and recurrent cases of MCD may imply a role for humoral immunity in its pathogenesis or communication between B and T cells [19–21]. SLE is characterized by polyclonal B cell activation and hyperreactivity along with T helper cell expansion [22] that hypothetically could contribute to the production of a permeability factor. Furthermore, interferon alpha, which has been identified as a participant in the pathogenetic pathway of SLE [23], when used as a treatment modality, has been reported in a few instances to be associated with MCD [24, 25]. Finally, complement-mediated injury, a major player in SLE disease, has been found able to disrupt the actin microfilament adhesion of podocytes to focal contacts and mediate their effacement [26]. In this context, the contribution of SLE in the development of MCD of this renal transplant with impaired venous outflow may also be interesting to explore.

Case reports or series have described the appearance of MCD in patients with a primary diagnosis of SLE. Dube et al. reported 7 cases of SLE patients with biopsy-proven MCD, of which 5 also exhibited mesangial electron-dense deposits with or without mesangial proliferation (lupus nephritis class II). All patients presented with nephrotic range proteinuria, peripheral edema, and hypoalbuminemia and responded to steroid therapy with rapid remission of their symptomatology. Three out of seven patients had a history of recent NSAID use [27]. Hertig et al. studied 11 SLE patients with nephrotic syndrome and a renal biopsy diagnosis of MCD in 4 patients or focal segmental glomerulosclerosis (FSGS) in 7 patients. None of the patients had a previous history or underlying lupus nephritis or triggers for secondary FSGS. Of interest was the fact that in nine out of eleven patients the development of nephrotic syndrome appeared at the time or shortly after SLE was diagnosed or coincided with a SLE flare of the disease. This observation led the authors to suggest that SLE could actively predispose to the development of nephrotic syndrome associated with MCD or FSGS [28]. We speculate that in our case iliac vein stenosis-induced increases of hydrostatic pressures, probably facilitated by a background of immune aberration, such as SLE, might have synergistically triggered the development of MCD.

*De novo* MCD in the posttransplantation period has been documented in the literature without, however, being attributed to any vascular complications of the graft. Although it has not been associated with any particular primary renal disease as a predisposing factor, it was observed in a case-series study that eight out of fourteen transplanted kidneys with* de novo* MCD originated from living donors, as seen in our patient. Moreover, most cases of posttransplantation nephrotic syndrome developed shortly after the surgery (within 4 months for 13/14 cases and at 24 months for one patient) and reached complete remission in 12/14 cases without adversely affecting in the long-term the kidney transplants [29].

Vascular complications after renal transplantation occur approximately in 2-3% of cases [30, 31]. The most common complication presented in large series of patients is renal artery stenosis whereas renal vein stenosis is fairly uncommon and mainly described in case reports [32–34] underlining the importance of recognizing such an entity in the clinical setting.

To our knowledge, this is a rare report of* de novo* MCD of a kidney transplant that is associated with iliac vein stenosis in the setting of SLE, which was reversible following stenting of the venous anastomosis. We suggest that in cases of nephrotic range proteinuria, especially with recurrent acute renal failure, without the other clinical features of the nephrotic syndrome, alternative causes of proteinuria, such as mechanical, should be considered, even with a biopsy-proven podocytopathy.

## Figures and Tables

**Table 1:** Laboratory findings 6 months after renal transplantation, at the start of proteinuria and creatinine rise.

CBC	

Hemoglobin (g/dL)	11.9
Hematocrit (%)	36.8
WBC (10^9^/L)	3.6
RBC (10^12^/L)	4.37
PLT (10^9^/L)	325
ANC (10^9^/L)	2.9
ALC (10^9^/L)	0.4

Metabolic panel	

Serum sodium (mmol/L)	136
Serum potassium (mmol/L)	4.3
Serum chloride (mmol/L)	107
Serum CO2 content (mmol/L)	20
Serum calcium (mg/dL)	9.3
Serum phosphorus (mmol/L)	3.5
Anion gap (mmol/L)	9
Serum creatinine (mg/dL)	1.48
Serum urea nitrogen (mg/dL)	22
Serum glucose (mg/dL)	86

Liver function tests	

Serum total protein (g/dL)	7
Serum albumin (g/dL)	4.4
Serum LDH (u/L)	178
Serum bilirubin total (mg/dL)	0.3
Serum bilirubin direct (mg/dL)	0.1
Serum ALP (u/L)	88
AST (u/L)	17
ALT (u/L)	12
INR	1
Prothrombin time (s)	10.3

Others	

Serum tacrolimus FK506 (ug/L)	7.7
Serum uric acid (mg/dL)	5.7
Creatine kinase (u/L)	143

SLE serology	

ANA by IF	Borderline
ANA titer/IF pattern	1 : 40/diffuse

Blood cultures	

Parvovirus B19	Not detected
Adenovirus Antibody	Not detected
BK virus PCR	Not detected
CMV virus PCR (copies/mL)	<200
EBV virus PCR (copies/mL)	<200

Urinalysis	

Urine color	Yellow
Urine appearance	Cloudy
Urine protein (mg/dL)	924
Urine creatinine (mg/dL)	202.8
RBCs	4–10
WBCs	10–25
Urine bacteria	Moderate
Urine blood	Negative
Urine ketones	Negative
Urine glucose	Negative
Urine pH	6
Urine bilirubin	Negative
Urine specific gravity	1.024
Urine nitrite	Negative
Urine leukocyte esterase	Negative
Urine culture	No growth of clean catch (<1,000 CFU/mL)
